# 7th *Eurosurveillance* scientific seminar ‘Artificial intelligence (AI) in epidemiology: a reality in 2018?’ at ESCAIDE 2018 in Malta

**DOI:** 10.2807/1560-7917.ES.2018.23.46.1811151

**Published:** 2018-11-15

**Authors:** Eurosurveillance editorial team

**Affiliations:** 1 European Centre for Disease Prevention and Control (ECDC), Stockholm, Sweden

**Keywords:** artificial intelligence, epidemiology

The 7th *Eurosurveillance *scientific seminar will be held as lunchtime seminar on 23 November 2018 during the ESCAIDE conference in Saint Julian's, Malta.

The seminar will feature a keynote lecture by Professor Magnus Boman, Royal Institute of Technology (KTH) and Research Institutes of Sweden (RISE), Stockholm, Sweden. Professor Boman will introduce general concepts related to artificial intelligence (AI) and illustrate related opportunities and challenges. He will also present existing and possible future AI applications in communicable disease epidemiology.

The European Commission has put AI high on its agenda and stressed that ‘to build robust models at the core of AI-based systems, high quality data are a key factor to improve performances’. To allow for innovation and to support the development of new technologies, revised rules on public sector information, as well as research and health data have been proposed to improve data sharing and open up more data for re-use while also taking into account interpretability and transparency which are needed to avoid AI systems becoming black boxes.

Professor Boman’s lecture will be followed by a panel discussion with epidemiologists and public health microbiologists. There will be room for our audience to take an active part in the discussion by sharing their experiences and expressing views.

The seminar will be open to all ESCAIDE participants. Due to the limited number of seats in the room, participants are welcome on a first come, first serve basis.

Read more about ESCAIDE here.

Read more about the Seminar here.

